# A Common Variant in SCN5A and the Risk of Ventricular Fibrillation Caused by First ST-Segment Elevation Myocardial Infarction

**DOI:** 10.1371/journal.pone.0170193

**Published:** 2017-01-13

**Authors:** Reza Jabbari, Charlotte Glinge, Javad Jabbari, Bjarke Risgaard, Bo Gregers Winkel, Christian Juhl Terkelsen, Hans-Henrik Tilsted, Lisette Okkels Jensen, Mikkel Hougaard, Stig Haunsø, Thomas Engstrøm, Christine M. Albert, Jacob Tfelt-Hansen

**Affiliations:** 1 Heart Center, Department of Cardiology, Copenhagen University Hospital, Rigshospitalet, Copenhagen, Denmark; 2 Department of Cardiology, Aarhus University Hospital, Skejby, Aarhus, Denmark; 3 Department of Cardiology, Aalborg University Hospital, Aalborg, Denmark; 4 Department of Cardiology, Odense University Hospital, Odense, Denmark; 5 Laboratory of Molecular Cardiology, Department of Cardiology, Copenhagen University Hospital, Rigshospitalet, Copenhagen, Denmark; 6 Center for Arrhythmia Prevention, Division of Preventive Medicine, Cardiovascular Division, Department of Medicine, Brigham and Women's Hospital, Harvard Medical School, Boston, Massachusetts, United States of America; Pennsylvania State University, UNITED STATES

## Abstract

**Background:**

Several common genetic variants have been associated with either ventricular fibrillation (VF) or sudden cardiac death (SCD). However, replication efforts have been limited. Therefore, we aimed to analyze whether such variants may contribute to VF caused by first ST-elevation myocardial infarction (STEMI).

**Methods:**

We analyzed 27 single nucleotide polymorphisms (SNP) previously associated with SCD/VF in other cohorts, and examined whether these SNPs were associated with VF caused by first STEMI in the GEnetic causes of Ventricular Arrhythmias in patients with first ST-elevation Myocardial Infarction (GEVAMI) study on ethnical Danes. The GEVAMI study is a prospective case-control study involving 257 cases (STEMI with VF) and 537 controls (STEMI without VF).

**Results:**

Of the 27 candidate SNPs, one SNP (rs11720524) located in intron 1 of *SCN5A which was previously* associated with SCD was significantly associated with VF caused by first STEMI. The major C-allele of rs11720524 was present in 64% of the cases and the C/C genotype was significantly associated with VF with an odds ratio (OR) of 1.87 (95% CI: 1.12–3.12; P = 0.017). After controlling for clinical differences between cases and controls such as age, sex, family history of sudden death, alcohol consumption, previous atrial fibrillation, statin use, angina, culprit artery, and thrombolysis in myocardial infarction (TIMI) flow, the C/C genotype of rs11720524 was still significantly associated with VF with an OR of 1.9 (95% CI: 1.05–3.43; P = 0.032). Marginal associations with VF were also found for rs9388451 in *HEY2* gene. The CC genotype showed an insignificant risk for VF with OR = 1.50 (95% CI: 0.96–2.40; P = 0.070).

**Conclusion:**

One common intronic variant in *SCN5A suggested an* association with VF caused by first STEMI. Further studies into the functional abnormalities associated with the noncoding variant in *SCN5A* may lead to important insights into predisposition to VF during STEMI.

## Introduction

Coronary artery disease and its ultimate consequence, myocardial infarction (MI), are believed to underlie 75% of the deaths of patients who experience sudden cardiac death (SCD).[1–3] It is also estimated that SCD accounts for 30 to 50% of all coronary deaths.[4] SCD is a major challenge for the clinician because most episodes occur in individuals without previously identified cardiac disease.[5,6] The pathophysiology of SCD caused by MI is complex. It is believed to involve an interaction between underlying disease and a transient event, such as acute myocardial ischemia, which results in electrical instability and ventricular fibrillation (VF).[2] VF is a life-threatening complication and occurs in nearly 12% of patients with first ST-segment elevation myocardial infarction (STEMI).[7,8] Therefore, efforts to improve risk stratification for SCD should in part be based on an understanding of the molecular mechanisms and pathways underlying the occurrence of VF.[9] In the **GE**netic causes of **V**entricular **A**rrhythmias in patients with first ST-elevation **M**yocardial **I**nfarction (GEVAMI) study on ethnical Danes[7] and the Dutch AGNES (**A**rrhythmia **G**enetics in the **NE**therlandS **S**tudy) study[10] both suggested a possible genetic component to VF since patients with a family history of sudden death among first-degree relatives had significantly higher odds of experiencing VF before primary percutaneous coronary intervention (PPCI). In the AGNES study, a common genetic variant at 21q21 (rs2824292, odds ratio (OR) = 1.78, 95% CI 1.47–2.13, P = 3.3x10^-10^) was found to be associated with VF before PPCI in STEMI patients in a genome-wide association study (GWAS).[11] The rs2824292 is located upstream of *CXADR* gene which encodes the coxsackie and adenovirus receptor (CAR), a transmembrane cell adhesion molecule predominantly located at the intercalated disc between cardiomyocytes.[12] However, this association has yet to be replicated in a larger cohort. There have also been several other variants reported to be associated with SCD, primarily from candidate gene studies with limited replication.[9] Therefore, we aimed to determine whether the 21q21variant, as well as other variants previously associated with SCD, would be associated with underlying susceptibility to VF due to first STEMI in the GEVAMI case-control study.

## Methods

### Study population

The GEVAMI study is an ongoing nationwide prospective Danish case-control study among patients with first STEMI between the ages of 18 and 80 years.[7] Cases are patients who experienced onset of VF within the first 12 hours of symptoms of STEMI before guided catheter insertion for PPCI, and controls did not have VF during this time period or during PPCI. Cases and controls are collected at all four PCI centers in Denmark. Cases and controls were required to have cardiac symptoms lasting ≤12 hours, acute STEMI on ECG, and a plan to proceed with for acute PPCI. Baseline demographics and previous medical history are collected by research coordinators utilizing pre-designed questionnaires and whole blood is collected for genetic analysis. Follow-up on the patients is done by the Danish registries.

### Ethics statement

Signed informed consent is available for all patients enrolled in this study. Procedures are in accordance with the ethical standards of the national ethics committee (protocol number: H-3-2010-133) on human research and with the Helsinki Declaration of 1975, as revised in 1983. Permission from the Danish Data Protection Agency was also obtained before the study was initiated (Jr.nr. 2010-41-5688). The study was conducted according to the guidelines of The National Committee on Health Research Ethics, Denmark.

### DNA extraction and SNP genotyping

In brief, total genomic DNA was isolated from whole blood samples using LGC’s Kleargene^™^ silica-based DNA extraction, performed at LGC Genomics. Isolated DNA was analyzed using UV spectrophotometry to estimate both the quality and quantity of the DNA and normalized.

Based upon the results of previous genetic association studies, 27 common genetic variants known to associate with VF/SCD[2,9,11,13–16,16–25] were selected for SNP genotyping in the cases and controls. SNP genotyping was performed using KASP^™^ genotyping assays from LGC genomics (http://www.lgcgenomics.com). For each putative varietal SNP, two allele-specific forward primers and one common reverse primer were designed (LGC Genomics, Hoddesdon, UK). KASP genotyping assays are based on competitive allele-specific PCR and enable biallelic scoring of SNPs at specific loci. The SNP-specific KASP Assay mix and the universal KASP Master mix are added to DNA samples, a thermal cycling reaction is then performed, followed by an end-point fluorescent read. Bi-allelic discrimination is achieved through the competitive binding of two allele-specific forward primers, each with a unique tail sequence that corresponds with two universal FRET (fluorescence resonant energy transfer) cassettes; one labelled with FAM^™^ dye and the other with HEX^™^ dye. All assays were conducted without knowledge of case status, and samples were labeled by study code only. Genotypes for all SNPs passed our quality-control threshold (call-rate ≥94%; Hardy-Weinberg equilibrium P>0.05 in control subjects).

### Statistical analysis

Medians or proportions of baseline and presenting characteristics in Table 1 were computed for cases and controls, and significance of associations were tested using the Wilcoxon rank-sum test for continuous variables and the χ2 test or Fisher exact test (where appropriate) for categorical variables. A two-tailed p value ≤0.05 was considered statistically significant. An age and sex-adjusted logistic regression model were constructed to estimate OR for the association between each SNP and VF using an additive model of inheritance. Any SNPs which were significantly associated with VF were also adjusted for clinical differences between cases and controls such as family history of sudden death, alcohol consumption, previous atrial fibrillation, statin use, angina, culprit artery, and thrombolysis in myocardial infarction (TIMI) flow.[7] We considered each SNP as a single hypothesis however a Bonferroni correction for multiple testing was used to assess the association of single SNP with VF. Homozygote risk allele and adjusted odds ratios are presented in Table 2. Per copy risk allele frequency is provided in Table 3. All analyses were performed using the Stata software package version 12.0 (StataCorp).

**Table 1:** Baseline characteristics of the cohort.

Variables		Cases (n = 257)	Controls (n = 537)	P-value
**Cardiovascular risk profile**				
Female sex, No. (%)		35 (14)	131 (25)	0.001
Median Age at index infarction, y (IQR^†^)		60 (53–68)	61 (52–66)	0.100
Body mass index (kg/m^2^), (IQR)		27.2 (25–29)	26.7 (24–29)	0.400
Smoking (pack year), (IQR)		25 (5–41)	25 (6–42)	0.200
Smoking, No. (%)				
	Never	38 (16)	108 (20)	0.300
	Past	69 (28)	133 (25)	
	Current	136 (56)	290 (55)	
Alcohol per week, (unit^‡^, IQR)		6 (1–15)	3 (0–9)	<0.001
Alcohol units per week (categorized), No. (%)				
	Non-drinkers	46 (19)	143 (27)	<0.001
	Normal (1–7)	90 (38)	242 (46)	
	Moderate High (8–14)	41 (17)	70 (13)	
	High (>15)	60 (26)	73 (14)	
Diabetes, No. (%)		30 (12)	47 (9)	0.200
Hypertension, No. (%)		102 (41)	184 (35)	0.070
COPD^§^, No. (%)		12 (5)	30 (6)	0.700
Hypercholesterolemia, No. (%)		97 (39)	165 (31)	0.023
Stroke, No. (%)		18 (7)	26 (5)	0.200
Atrial fibrillation, No. (%)		16 (7)	10 (2)	0.006
Depression, No. (%)		28 (11)	65 (12)	0.700
Epilepsy, No. (%)		4 (2)	5 (1)	0.500
**Family History of, No. (%)**				
	Sudden death	94 (40%)	128 (25%)	<0.001
	Myocardial infarction	90 (40%)	195 (38%)	0.600
	Stroke	36 (16%)	75 (15%)	0.600
**Medication before STEMI, No. (%)**				
	β-blockers	20 (8)	43 (8)	0.900
	Statins	55 (22)	65 (12)	<0.001
	ACE/ARB^#^ blockers	50 (21)	90 (17)	0.200
	Aspirin	28 (12)	40 (8)	0.060
**Procedural characteristics, No. (%)**				
Infarct location				
	Anterior	136 (57)	103 (43)	0.001
	Non-anterior	213 (43)	280 (57)	
**Preprocedural TIMI flows, No. (%)**				
	TIMI 0	137 (57)	240 (49)	0.017
	TIMI I	17 (7)	50 (10)	
	TIMI II	23 (9)	83 (17)	
	TIMI III	64 (27)	117 (24)	
**Postprocedural TIMI flows, No. (%)**				
	TIMI 0	8 (3)	7 (1)	0.080
	TIMI I	0 (0)	0 (0)	
	TIMI II	12 (5)	14 (3)	
	TIMI III	220 (92)	469 (96)	
**Cardiac Symptoms Within 1 Year Prior to STEMI, No. (%)**				
	Angina	117 (49)	313 (64)	<0.001
	Dyspnea	55 (24)	127 (26)	0.500
	Palpitations	17 (7)	46 (9)	0.400
	Syncope	4 (2)	5(1)	0.500

IQR^†^: interquartile range; unit^‡^ of alcohol = 12gram (1 drink); COPD^§^: chronic obstructive pulmonary disease; ACE/ARB^#^: angiotensin-converting-enzyme inhibitor/ angiotensin II receptor blocker. TIMI: thrombolysis in myocardial infarction

**Table 2:** Homozygote genotype of inheritance for association of 27 SNPs previously associated with sudden cardiac death and in this study investigated for association with ventricular fibrillation before ST-segment elevation myocardial infarction.

	Chr^†^	SNP^‡^	Nearest Gene	Homozygote Risk Allele	OR^§^	95% CI*	P-value^#^
1	1	rs10918859	*NOS1AP*	AA	1.01	0.47–2.18	0.970
2	1	rs12084280	*NOS1AP*	CC	1.90	0.26–13.70	0.500
3	1	rs16847548	*NOS1AP*	CC	0.86	0.38–1.93	0.720
4	1	rs17500488	*CASQ2*	CC	1.13	0.10–13.04	0.920
5	1	rs3010396	*CASQ2*	AA	0.89	0.57–1.38	0.600
6	1	rs7366407	*CASQ2*	AA	0.71	0.33–1.52	0.380
7	1	rs12090554	*HMCN1*	CC	0.76	0.34–1.68	0.500
8¶	2	rs4665058	*BAZ2B*	AA	**-**	**-**	**-**
9	2	rs6730157	*RAB3GAP1*	GG	1.43	0.80–2.57	0.220
10	3	**rs11720524**	***SCN5A***	**CC**	**1.87**	**1.12–3.12**	**0.017**
11	3	rs11708996	*SCN5A*	CC	1.03	0.40–2.63	0.940
12	3	rs41312391	*SCN5A*	AA	0.39	0.13–1.17	0.094
13¶	3	rs6795970	*SCN10A*	AA	-	-	-
14	3	rs10428132	*SCN10A*	TT	1.00	0.61–1.61	0.990
15	3	rs9862154	*GPD1L*	GG	1.03	0.47–2.28	0.920
16¶	4	rs2200733	*PITX2*	TT	**-**	**-**	**-**
17¥	5	rs7737692	*LPCAT1*	GG	**-**	**-**	**-**
18	5	rs1042714	*ADRB2*	GG	0.97	0.62–1.52	0.913
19	6	**rs9388451**	***HEY2***	**CC**	**1.50**	**0.96–2.40**	**0.070**
20	8	rs10503929	*NRG1*	CC	0.92	0.39–2.16	0.850
21	9	rs10757274	*CDKN2A/2B*	GG	1.15	0.73–1.81	0.525
22	9	rs2383207	*CDKN2A/2B*	AA	0.76	0.48–1.20	0.250
23	9	rs1353342	*PCSK5*	AA	2.73	0.60–12.40	0.190
24	10	rs2077316	*ZNF365*	GG	1.44	0.23–8.88	0.690
25	11	rs2283222	*KCNQ1*	CC	1.48	0.90–2.40	0.120
26	13	rs3864180	*GPC5*	GG	0.68	0.36–1.03	0.130
27	21	rs2824292	*CXADR*	GG	0.77	0.47–1.26	0.300

Chr^†^: chromosome; SNP^‡^: single-nucleotide polymorphism; OR§: odds ratio; CI*: confidence interval; P value^#^ for the additive genetic model of inheritance.

^**¶**^: No homozygote risk allele exists in our cohort.

^**¥**^: No signal at all (repeated 3 times). Logistic regression models under an additive model of inheritance adjusted for age and sex. OR for the homozygote risk allele is shown in the table. Number of cases = 257; number of controls = 537.

**Table 3:** Additive genetic model of inheritance (per copy allele frequency) for association of 27 SNPs previously associated with sudden cardiac death, and in this study investigated for association with ventricular fibrillation before ST-segment elevation myocardial infarction.

	Chr^†^	SNP^‡^	Nearest Gene (References)	RAF^§^	Associated Allele Frequency (Cases/Controls)	OR*	95% CI^#^	P-value
1	1	rs10918859	*NOS1AP*[24]	A	0.17/0.20	0.78	0.59–1.04	0.090
2	1	rs12084280	*NOS1AP*[24]	C	0.07/0.08	0.94	0.62–1.41	0.800
3	1	rs16847548	*NOS1AP*[22]	C	0.21/0.22	0.93	0.71–1.22	0.600
4	1	rs17500488	*CASQ2*[24]	C	0.07/0.07	1.10	0.72–1.65	0.700
5	1	rs3010396	*CASQ2*[24]	A	0.43/0.45	0.93	0.75–1.17	0.600
6	1	rs7366407	*CASQ2*[24]	A	0.23/0.26	0.90	0.67–1.20	0.500
7	1	rs12090554	*HMCN1*[11]	C	0.18/0.20	0.88	0.67–1.16	0.400
8¶	2	rs4665058	*BAZ2B*[17]	A	0.00/0.00	**-**	**-**	**-**
9	2	rs6730157	*RAB3GAP1*[21]	G	0.26/0.23	1.12	0.88–1.43	0.400
10	3	**rs11720524**	***SCN5A***[14]	**C**	**0.64/0.58**	**1.25**	**0.99–1.57**	**0.050**
11	3	rs11708996	*SCN5A*[13]	C	0.15/0.16	1.00	0.75–1.34	1.000
12	3	rs41312391	*SCN5A*[25]	A	0.17/0.18	0.94	0.70–1.25	0.700
13¶	3	rs6795970	*SCN10A*[13]	A	0.00/0.00	-		
14	3	rs10428132	*SCN10A*[13]	T	0.37/.038	1.00	0.90–1.24	0.900
15	3	rs9862154	*GPD1L*[24]	G	0.22/0.20	1.10	0.85–1.45	0.400
16¶	4	rs2200733	*PITX2*[25]	T	0.08/0.08	0.96	0.65–1.42	0.900
17¥	5	rs7737692	*LPCAT1*[19]	G	**-/-**	**-**	**-**	**-**
18	5	rs1042714	*ADRB2*[15,23]	G	0.42/0.42	1.01	0.81–1.25	0.900
19	6	**rs9388451**	***HEY2***[13]	**C**	**0.54/0.49**	**1.20**	**0.97–1.50**	**0.090**
20	8	rs10503929	*NRG1*[20]	C	0.18/0.20	0.92	0.69–1.20	0.500
21	9	rs10757274	*CDKN2A/2B*[16]	G	0.50/0.49	1.08	0.86–1.35	0.500
22	9	rs2383207	*CDKN2A/2B*[16]	A	0.45/0.48	0.87	0.70–1.10	0.300
23	9	rs1353342	*PCSK5*[11]	A	0.12/0.10	1.14	0.80–1.66	0.400
24	10	rs2077316	*ZNF365*[21]	G	0.06/0.07	0.97	0.63–1.49	0.900
25	11	rs2283222	*KCNQ1*[14]	C	0.34/0.30	1.18	0.94–1.47	0.100
26	13	rs3864180	*GPC5*[18]	G	0.37/0.40	0.89	0.71–1.10	0.300
27	21	rs2824292	*CXADR*[11]	G	0.49/0.54	0.93	0.75–1.18	0.600

Chr^†^: chromosome; SNP^‡^: single-nucleotide polymorphism; RAF^§^: Risk allele frequency in cases over controls in our cohort; OR*: odds ratio; CI^#^: confidence interval; P value for the additive genetic model of inheritance (per copy allele frequency). Logistic regression models under an additive model of inheritance adjusted for age and sex. Number of cases = 257; number of controls = 537.

^**¶**^: No homozygote risk allele exists in our cohort.

^**¥**^: No signal at all (repeated 3 times).

## Results

### Clinical characteristics of the cohort

A total of 257 cases (35 women and 222 men) with VF caused by STEMI and 537 (131 women and 426 men) STEMI controls without VF were included in this prospective study. The clinical characteristics at the time of STEMI are shown in Table 1. The median age of the cases was 60 (interquartile range (IQR); 53–68) years and for the controls 61 (IQR; 52–66) years. Compared to the control group, the case group was more likely to be male, have a history of atrial fibrillation (AF) or hypercholesterolemia, and a family history of sudden death as reported previously.[7] Furthermore, the proportion on statin therapy was higher in the case group compared to the control group, likely due to the higher degree of hypercholesterolemia in the case group (Table 1). Median levels of average weekly alcohol intake were higher in the case group (6 units/week) as compared to the control group (3 units/week) without VF (P = 0.001). Cases did not differ significantly from the controls with respect to other cardiovascular risk factors such as smoking, diabetes, and hypertension.

### Genotyping

A panel of 27 SNPs was genotyped in 794 individuals (Table 2). The overall call rate was ≥94%. Homozygote risk allele and the results of the case-control association are presented in Table 2. The strongest association was observed for the rs11720524 SNP which is located in intron 1 of *SCN5A* near the promoter region. The homozygous C/C genotype of rs11720524 was associated with VF with an OR of 1.87 (95% CI: 1.12–3.12; P = 0.017) (Table 3). After controlling for clinical differences between cases and controls such as age, sex, family history of sudden death, alcohol consumption, previous atrial fibrillation, statin use, angina, culprit artery, and thrombolysis in myocardial infarction (TIMI) flow, the C/C genotype of rs11720524 was still significantly associated with VF with an OR of 1.9 (95% CI: 1.05–3.43; P = 0.032) (not shown in the table). Even though we tested each SNP as a single hypothesis, the rs11720524 SNP was not associated with VF after Bonferroni correction.

The risk allele frequencies for each SNP and the results from logistic regression analysis adjusted for age and sex under additive genetic model of inheritance (per copy allele frequency) are presented in Table 3. Each increasing copy of the major C-allele (per allele risk) of rs11720524 was borderline associated with VF with an OR of 1.25 (95% CI: 0.99–1.57; P = 0.050) (Table 3). The call rate for the rs11720524 SNP was 94.2%.

Furthermore, the rs9388451 SNP which is a non-coding SNP and is near the HEY2 gene showed trend for association with VF. Each increasing copy of the risk allele C-allele (per allele risk) of rs9388451 was borderline associated with VF with an OR of 1.20 (95% CI: 0.97–1.50; P = 0.090) (Table 2) and the homozygous CC genotype showed an insignificant increase risk for VF with OR = 1.50 (95% CI: 0.96–2.40; P = 0.070) (Table 3).

The common genetic variant at 21q21 (rs2824292) previously associated with VF before PPCI in STEMI patients in the AGNES study[11] was not replicated in the present GEVAMI study (OR = 0.93; 95% CI: 0.75–1.18; P = 0.600) (Table 2).

For two SNPs (rs4665058 and rs6795970), a homozygote risk allele (A/A and G/G, respectively) did not exists in our cohort although the average call-rate was 97.8% for the two SNPs. We were not able to genotype the SNP rs7737692 even after 3 times replication. No other SNPs were associated with VF (Tables 2 and 3).

## Discussion

In this prospective case-control study in Denmark we identified a common variant (rs11720524) located in intron 1 of the *SCN5A* gene near the promoter region which was associated with VF in patients with STEMI. To our knowledge, this is the first study linking a common variant in *SCN5A*, the major sodium ion channel in the heart, to VF caused by first STEMI. Genetic studies have been successful in uncovering rare mutations in ion channel genes and these have been associated with rare Mendelian arrhythmic disorders, most notably the long-QT syndrome and to a lesser degree Brugada syndrome,[9,26] and for some of these disorders genetic testing has become a pivotal part of clinical care.[27,28] In contrast, studies aimed at identifying genetic risk factors for VF in the setting of MI affecting the majority and older segment of the population have been sparse[11] and therefore genetic architecture of this complex and multi-factorial disease largely remains unknown.

The major C allele of rs11720524 has previously been shown associated to SCD in a combined nested case-control analysis among 516 cases and 1522 matched control subjects of European ancestry enrolled in 6 prospective cohort studies.[14] Although the associations between the SNP (rs11720524) and sudden/arrhythmic death were relatively consistent across the 6 studies, nevertheless this finding required an independent replication study as it was done in our case-control setup which has a comparable size and is well-phenotyped (VF), which is necessary to establish certainty regarding the observed association. To support the association of rs11720524 with VF in our study a recent study also showed an association of the C/C genotype of the rs11720524 with SCD (OR = 1.351; p = 0.019).[29] Although this association was shown in a highly heterogeneous cardiac death cohort further subgroup analysis also showed an association of the C/C genotype of the rs11720524 with SCD in chronic ischemic heart disease patients (OR = 1.455; p = 0.012). Two other SNPs in *SCN5A* (rs41312391 and rs11708996) were not associated to VF in the GEVAMI cohort. The SNP rs41312391risk allele (A) which is also located in the intron near *SCN5A* has been associated with QT-interval prolongation in normal subjects in one study (n = 282, P = 0.04).[30] and QT-interval shortening in another study (n = 396, P = 0.02).[31] Furthermore, in the FinSCDgen Study, Lahtinen et al. and coworkers showed an association of the SNP rs41312391 risk allele (A) with SCD with relative risk 1.27 per minor T allele, 95% CI 1.11–1.45, p = 3.4x10^-4^).[25] The expression analysis indicated that rs41312391 may change the expression level of a nearby gene, *WDR48*, which encodes a WD repeat-containing protein, a regulator of histone deubiquitinating complexes.[25] However, they concluded that *SCN5A* remains the more likely candidate gene for SCD due to its known function in cardiac conduction and repolarization. Furthermore, the SNP rs11708996 has been associated with PR interval (which is an intermediate phenotype of atrial fibrillation),[32] QT interval[33] and Brugada syndrome.[34] This finding suggests that the SNP rs11708996 modifying effects are distinct and in the GEVAMI cohort did not predict risk of VF in STEMI patients.

Identifying the exact causal SNP within the haplotype tagged by (rs11720524) to understand the underlying genetic mechanism is important and requires functional studies to further understand the possible role of this gene in the setting of VF. Since SNPs are highly correlated (in linkage) to the neighboring SNPs and thereby forming haplotypes and presumably these intronic variants in this halpotypes may exert their influence through the level of expression of *SCN5A*. A number of SNPs at the *SCN5A* and *SCN10A* locus (Table 2) have been associated with ECG changes such as QT, RR and QRS interval[9], however our lead SNP (rs11720524) was not in linkage disequilibrium with any one of these.

The rs9388451 SNP is a non-coding SNP and near the *HEY2* gene and encodes a basic helix-loop-helix transcriptional repressor that is expressed in the cardiovascular system.[35] Recently, the rs9388451 SNP was shown to be associated with Brugada syndrome and data indicates that *HEY2* regulates cardiac electrical activity in Brugada syndrome by altering the transcriptional programming during cardiac development.[13] Even though the association of the (C/C) rs9388451 was not significant (OR = 1.50; P = 0.070), it showed the same trend and it could be due to lack of power in our analysis.

Several studies[7,10,36] such as our Danish GEVAMI study and the Dutch AGNES study, showed an association of family history of sudden death with VF caused by first STEMI suggesting a genetic burden of the disease. The AGNES study reported the first GWAS for STEMI patients with VF before PPCI. In this GWAS study the most significant association with VF was found at 21q21 (rs2824292, OR = 1.78, 95% CI:1.47–2.13; P = 3.3x10^-10^).[11] Despite this novel and important finding, the association of SNP rs2824292 was not detected in the GEVAMI study nor in a small German case-control study.[7,37] However, there is no obvious explanation for this disparity. It could be explained by lack of power or maybe the effect of the risk allele may differ between populations because of gene-gene or gene-environment interactions.[38] Larger sample sizes and homogenous enriched subgroups will be needed to identify and replicate additional genetic variants associated with VF during STEMI. For instance the location of the culprit coronary lesion may modify QRS duration and QTc interval and thereby risk of VF.[9,39] Stratification of VF patients based on culprit lesion may give us a more discrete phenotype, but this requires a very large sample size. An even more discrete phenotype based on the mechanism of VF (whether it is an immediate VF (phase Ia) or delayed (after the first 15 minutes) VF (phase Ib)) may also be warranted.[40] This hypothesis is supported by experimental data comparing wild-type (WT) and heterozygous CAR deficient (CAR^+/−^) mice.[39] These data suggested that the burden of VF is much higher in CAR^+/−^ mice with acute left anterior descending (LAD) occlusion compared to the WT, and this higher VF burden was only observed within the first 15 minutes of acute occlusion of LAD. During later stages of the ischemic episode, arrhythmia inducibility was not different between WT and CAR^+/−^ mice.[39]

Lastly, in a combined meta-analysis of GWAS studies of individuals with SCD and control individuals of European ancestry, a locus at chromosome 2q24.2 (rs4665058) was found to be associated with SCD[17]; however, this SNP was not associated to VF in the AGNES study or in the GEVAMI study. These inconsistencies could be caused by heterogeneous underlying cardiac pathologies of SCD and differing phenotypes (i.e., VF vs. SCD).[9]

The common SNPs associated with VF in the studies mentioned above all by nature had small effect sizes on risk and are not currently used for clinical management. With the rapid development of next*-*generation sequencing technologies, large-scale sequencing projects are becoming possible allowing the examination of rare genetic variants with larger effects which could be useful for risk stratification.

We considered each SNP as a single hypothesis but a Bonferroni correction for multiple testing is often applied in genetic association studies but might be too conservative for highly selective candidate SNP-based approaches. However, we need to acknowledge that none of the SNPs would be significant if Bonferroni corrected for multiple testing (P = 0.0019[0.05/27]). Furthermore, the sample size of 257 VF cases is still fairly low and limits the ability of this study to find association of common variants with modest effect. It is also important to mention that collecting cases is very time consuming and limited due to relatively low incidence of VF and difficulties to enroll and collect blood samples in STEMI-patients with VF and cardiac arrest. This is mostly due to that patients who died outside of the hospital or died in-hospital prior to enrollment cannot be included. Most of the 27 SNPs reported to be associated with SCD/VF were identified in patients without acute coronary syndrome, hence may reflect the underlying pathophysiology of SCD/VF in a more general population rather patients with VF due to first STEMI.

Finally, our results in this population with white, European/Danish ancestry may not be generalizable to other populations.

The present study nicely shows that it is often difficult to replicate the genetic associations observed in previous studies in some other populations. The genetic factors involved in this complex and multi-factorial disease are still largely unknown; therefore the current study on all the previous SNPs associated with SCD/VF is important, especially for future direction of functional studies for more clarification of arrhythmia mechanism and targeted intervention strategies.

## References

[1] de Vreede-Swagemakers JJ, Gorgels AP, Dubois-Arbouw WI, van Ree JW, Daemen MJ, Houben LG, et al Out-of-hospital cardiac arrest in the 1990’s: a population-based study in the Maastricht area on incidence, characteristics and survival. J Am Coll Cardiol. 1997;30: 1500–1505. 9362408 10.1016/s0735-1097(97)00355-0

[2] Deo R, Albert CM. Epidemiology and genetics of sudden cardiac death. Circulation. 2012;125: 620–637. 10.1161/CIRCULATIONAHA.111.023838 22294707 PMC3399522

[3] Jabbari R. Ventricular fibrillation and sudden cardiac death during myocardial infarction. Dan Med J. 2016;63. 27127021

[4] Gillum RF. Sudden coronary death in the United States: 1980–1985. Circulation. 1989;79: 756–765. 2924409 10.1161/01.cir.79.4.756

[5] Huikuri HV, Castellanos A, Myerburg RJ. Sudden death due to cardiac arrhythmias. N Engl J Med. 2001;345: 1473–1482. 10.1056/NEJMra000650 11794197

[6] Jabbari R, Risgaard B, Holst AG, Nielsen JB, Glinge C, Engstrøm T, et al Cardiac symptoms before sudden cardiac death caused by coronary artery disease: a nationwide study among young Danish people. Heart. 2013;99: 938–943. 10.1136/heartjnl-2012-303534 23574972

[7] Jabbari R, Engstrøm T, Glinge C, Risgaard B, Jabbari J, Winkel BG, et al Incidence and risk factors of ventricular fibrillation before primary angioplasty in patients with first ST-elevation myocardial infarction: a nationwide study in Denmark. J Am Heart Assoc. 2015;4: e001399 10.1161/JAHA.114.001399 25559012 PMC4330064

[8] Jabbari R, Risgaard B, Fosbøl EL, Scheike T, Philbert BT, Winkel BG, et al Factors Associated With and Outcomes After Ventricular Fibrillation Before and During Primary Angioplasty in Patients With ST-Segment Elevation Myocardial Infarction. Am J Cardiol. 2015;116: 678–685. 10.1016/j.amjcard.2015.05.037 26150175

[9] Marsman RF, Tan HL, Bezzina CR. Genetics of sudden cardiac death caused by ventricular arrhythmias. Nat Rev Cardiol. 2014;11: 96–111. 10.1038/nrcardio.2013.186 24322550

[10] Dekker LRC, Bezzina CR, Henriques JPS, Tanck MW, Koch KT, Alings MW, et al Familial sudden death is an important risk factor for primary ventricular fibrillation: a case-control study in acute myocardial infarction patients. Circulation. 2006;114: 1140–1145. 10.1161/CIRCULATIONAHA.105.606145 16940195

[11] Bezzina CR, Pazoki R, Bardai A, Marsman RF, de Jong JSSG, Blom MT, et al Genome-wide association study identifies a susceptibility locus at 21q21 for ventricular fibrillation in acute myocardial infarction. Nat Genet. 2010;42: 688–691. 10.1038/ng.623 20622880 PMC3966292

[12] Noutsias M, Fechner H, de Jonge H, Wang X, Dekkers D, Houtsmuller AB, et al Human coxsackie-adenovirus receptor is colocalized with integrins alpha(v)beta(3) and alpha(v)beta(5) on the cardiomyocyte sarcolemma and upregulated in dilated cardiomyopathy: implications for cardiotropic viral infections. Circulation. 2001;104: 275–280. 11457744 10.1161/01.cir.104.3.275

[13] Bezzina CR, Barc J, Mizusawa Y, Remme CA, Gourraud J-B, Simonet F, et al Common variants at SCN5A-SCN10A and HEY2 are associated with Brugada syndrome, a rare disease with high risk of sudden cardiac death. Nat Genet. 2013;45: 1044–1049. 10.1038/ng.2712 23872634 PMC3869788

[14] Albert CM, MacRae CA, Chasman DI, VanDenburgh M, Buring JE, Manson JE, et al Common variants in cardiac ion channel genes are associated with sudden cardiac death. Circ Arrhythm Electrophysiol. 2010;3: 222–229. 10.1161/CIRCEP.110.944934 20400777 PMC2891421

[15] Gavin MC, Newton-Cheh C, Gaziano JM, Cook NR, VanDenburgh M, Albert CM. A common variant in the β2-adrenergic receptor and risk of sudden cardiac death. Heart Rhythm Off J Heart Rhythm Soc. 2011;8: 704–710. 10.1016/j.hrthm.2011.01.003 PMC3225286 21215823

[16] Newton-Cheh C, Cook NR, VanDenburgh M, Rimm EB, Ridker PM, Albert CM. A common variant at 9p21 is associated with sudden and arrhythmic cardiac death. Circulation. 2009;120: 2062–2068. 10.1161/CIRCULATIONAHA.109.879049 19901189 PMC2785227

[17] Arking DE, Junttila MJ, Goyette P, Huertas-Vazquez A, Eijgelsheim M, Blom MT, et al Identification of a sudden cardiac death susceptibility locus at 2q24.2 through genome-wide association in European ancestry individuals. PLoS Genet. 2011;7: e1002158 10.1371/journal.pgen.1002158 21738491 PMC3128111

[18] Arking DE, Reinier K, Post W, Jui J, Hilton G, O’Connor A, et al Genome-wide association study identifies GPC5 as a novel genetic locus protective against sudden cardiac arrest. PloS One. 2010;5: e9879 10.1371/journal.pone.0009879 20360844 PMC2845611

[19] Lemaitre RN, Johnson CO, Hesselson S, Sotoodehnia N, Sotoodhenia N, McKnight B, et al Common variation in fatty acid metabolic genes and risk of incident sudden cardiac arrest. Heart Rhythm Off J Heart Rhythm Soc. 2014;11: 471–477. 10.1016/j.hrthm.2014.01.008 PMC3966996 24418166

[20] Huertas-Vazquez A, Teodorescu C, Reinier K, Uy-Evanado A, Chugh H, Jerger K, et al A common missense variant in the neuregulin 1 gene is associated with both schizophrenia and sudden cardiac death. Heart Rhythm Off J Heart Rhythm Soc. 2013;10: 994–998. 10.1016/j.hrthm.2013.03.020 PMC3692570 23524320

[21] Huertas-Vazquez A, Nelson CP, Guo X, Reinier K, Uy-Evanado A, Teodorescu C, et al Novel loci associated with increased risk of sudden cardiac death in the context of coronary artery disease. PloS One. 2013;8: e59905 10.1371/journal.pone.0059905 23593153 PMC3617189

[22] Kao WHL, Arking DE, Post W, Rea TD, Sotoodehnia N, Prineas RJ, et al Genetic variations in nitric oxide synthase 1 adaptor protein are associated with sudden cardiac death in US white community-based populations. Circulation. 2009;119: 940–951. 10.1161/CIRCULATIONAHA.108.791723 19204306 PMC2782762

[23] Sotoodehnia N, Siscovick DS, Vatta M, Psaty BM, Tracy RP, Towbin JA, et al Beta2-adrenergic receptor genetic variants and risk of sudden cardiac death. Circulation. 2006;113: 1842–1848. 10.1161/CIRCULATIONAHA.105.582833 16618831

[24] Westaway SK, Reinier K, Huertas-Vazquez A, Evanado A, Teodorescu C, Navarro J, et al Common variants in CASQ2, GPD1L, and NOS1AP are significantly associated with risk of sudden death in patients with coronary artery disease. Circ Cardiovasc Genet. 2011;4: 397–402. 10.1161/CIRCGENETICS.111.959916 21685173 PMC3160237

[25] Lahtinen AM, Noseworthy PA, Havulinna AS, Jula A, Karhunen PJ, Kettunen J, et al Common genetic variants associated with sudden cardiac death: the FinSCDgen study. PloS One. 2012;7: e41675 10.1371/journal.pone.0041675 22844511 PMC3402479

[26] Tfelt-Hansen J, Winkel BG, Grunnet M, Jespersen T. Inherited cardiac diseases caused by mutations in the Nav1.5 sodium channel. J Cardiovasc Electrophysiol. 2010;21: 107–115. 10.1111/j.1540-8167.2009.01633.x 19845816

[27] Priori SG, Wilde AA, Horie M, Cho Y, Behr ER, Berul C, et al HRS/EHRA/APHRS Expert Consensus Statement on the Diagnosis and Management of Patients with Inherited Primary Arrhythmia Syndromes: Document endorsed by HRS, EHRA, and APHRS in May 2013 and by ACCF, AHA, PACES, and AEPC in June 2013. Heart Rhythm. 2013;10: 1932–1963. 10.1016/j.hrthm.2013.05.014 24011539

[28] Wilde AAM, Behr ER. Genetic testing for inherited cardiac disease. Nat Rev Cardiol. 2013;10: 571–583. 10.1038/nrcardio.2013.108 23900354

[29] Marcsa B, Dénes R, Vörös K, Rácz G, Sasvári-Székely M, Rónai Z, et al A Common Polymorphism of the Human Cardiac Sodium Channel Alpha Subunit (SCN5A) Gene Is Associated with Sudden Cardiac Death in Chronic Ischemic Heart Disease. PloS One. 2015;10: e0132137 10.1371/journal.pone.0132137 26146998 PMC4492622

[30] Aydin A, Bähring S, Dahm S, Guenther UP, Uhlmann R, Busjahn A, et al Single nucleotide polymorphism map of five long-QT genes. J Mol Med Berl Ger. 2005;83: 159–165. 10.1007/s00109-004-0595-3 15599693

[31] Gouas L, Nicaud V, Chaouch S, Berthet M, Forhan A, Tichet J, et al Confirmation of associations between ion channel gene SNPs and QTc interval duration in healthy subjects. Eur J Hum Genet EJHG. 2007;15: 974–979. 10.1038/sj.ejhg.5201866 17534376 PMC2234597

[32] Pfeufer A, van Noord C, Marciante KD, Arking DE, Larson MG, Smith AV, et al Genome-wide association study of PR interval. Nat Genet. 2010;42: 153–159. 10.1038/ng.517 20062060 PMC2850197

[33] Sotoodehnia N, Isaacs A, de Bakker PIW, Dörr M, Newton-Cheh C, Nolte IM, et al Common variants in 22 loci are associated with QRS duration and cardiac ventricular conduction. Nat Genet. 2010;42: 1068–1076. 10.1038/ng.716 21076409 PMC3338195

[34] Bezzina CR, Barc J, Mizusawa Y, Remme CA, Gourraud J-B, Simonet F, et al Common variants at SCN5A-SCN10A and HEY2 are associated with Brugada syndrome, a rare disease with high risk of sudden cardiac death. Nat Genet. 2013;45: 1044–1049. 10.1038/ng.2712 23872634 PMC3869788

[35] Leimeister C, Externbrink A, Klamt B, Gessler M. Hey genes: a novel subfamily of hairy- and Enhancer of split related genes specifically expressed during mouse embryogenesis. Mech Dev. 1999;85: 173–177. 10415358 10.1016/s0925-4773(99)00080-5

[36] Jouven X, Desnos M, Guerot C, Ducimetière P. Predicting sudden death in the population: the Paris Prospective Study I. Circulation. 1999;99: 1978–1983. 10209001 10.1161/01.cir.99.15.1978

[37] Bugert P, Elmas E, Stach K, Weiss C, Kälsch T, Dobrev D, et al No evidence for an association between the rs2824292 variant at chromosome 21q21 and ventricular fibrillation during acute myocardial infarction in a German population. Clin Chem Lab Med CCLM FESCC. 2011;49: 1237–1239. 10.1515/CCLM.2011.190 21574885

[38] Manolio TA, Collins FS, Cox NJ, Goldstein DB, Hindorff LA, Hunter DJ, et al Finding the missing heritability of complex diseases. Nature. 2009;461: 747–753. 10.1038/nature08494 19812666 PMC2831613

[39] Marsman RFJ, Bezzina CR, Freiberg F, Verkerk AO, Adriaens ME, Podliesna S, et al Coxsackie and adenovirus receptor is a modifier of cardiac conduction and arrhythmia vulnerability in the setting of myocardial ischemia. J Am Coll Cardiol. 2014;63: 549–559. 10.1016/j.jacc.2013.10.062 24291282 PMC3926969

[40] Janse MJ, Wit AL. Electrophysiological mechanisms of ventricular arrhythmias resulting from myocardial ischemia and infarction. Physiol Rev. 1989;69: 1049–1169. 2678165 10.1152/physrev.1989.69.4.1049

